# Comparative Study of Audiovestibular Symptoms between Early and Late Variants of COVID-19

**DOI:** 10.3390/audiolres12060065

**Published:** 2022-12-04

**Authors:** Ali A. Almishaal

**Affiliations:** Department of Speech-Language Pathology and Audiology, College of Applied Medical Sciences, University of Hail, Hail 55476, Saudi Arabia; a.almishaal@uoh.edu.sa

**Keywords:** COVID-19, SARS-CoV-2, Audiovestibular, hearing loss, tinnitus, vertigo, dizziness

## Abstract

Audiovestibular symptoms during the acute phase of the corona virus disease 2019 (COVID-19), have been reported for earlier waves of the pandemic, while no studies investigated nor compared audiovestibular manifestations during subsequent waves of COVID-19. In the current study, we aimed to compare the occurrence of audiovestibular symptoms associated with COVID-19 between the alpha/beta, delta, and omicron variants. An online questionnaire was distributed to individuals with confirmed test results for COVID-19. We asked participants to report whether they experienced audiovestibular symptoms during the acute phase of the disease. The study included 939 participants; 120 un-infected controls and infected participants during alpha/beta (n = 301), delta (n = 102), and omicron (n = 416) predominance periods. Self-reported audiovestibular symptoms were found to be statistically significantly different between un-infected controls and COVID-19 infected individuals in all analyzed variants. Furthermore, our results showed no significant differences in audiovestibular symptoms among individuals infected during alpha/beta, delta, and omicron waves. Although individuals infected during the delta variant predominance period reported higher percentages of audiovestibular symptoms (ranging from 11.8% to 26.5% for auditory symptoms and from 12.7% to 34.3% for vestibular symptoms) than for the alpha/beta (ranging from 6.3% to 18.9% for auditory symptoms and 8.3% to 29.9% for vestibular symptoms) and omicron (ranging from 9.6% to 21.2% for auditory and 12.5 to 29.1% for vestibular symptoms) variants, this did not achieve statistical significance. With regards to auditory symptoms, the most commonly reported symptoms were aural fullness followed by hearing loss and tinnitus. With regards to vestibular symptoms, dizziness was the most commonly reported symptom followed by vertigo and unsteadiness. Logistic regression revealed that experiencing auditory symptoms were associated with other neurological symptoms, back and joint pain, and chest pain as COVID-19 symptoms. Vestibular symptoms were associated with anemia, gender, fatigue, headache, and breathing difficulties. In conclusion, our study shows that audiovestibular symptoms are common during the acute phase of early and late COVID-19 variants with no significant differences between them.

## 1. Introduction

Coronavirus disease 2019 (COVID-19), caused by the severe acute respiratory syndrome coronavirus 2 (SARS-CoV-2), first appeared in Wuhan, China in late 2019 and since has evolved into a global pandemic that has caused serious health threats and profoundly disrupted fundamental life activities. SARS-CoV-2 spreads through respiratory droplets via direct and close contact between infected, symptomatic and highly likely asymptomatic individuals. Transmission by indirect contact with contaminated surfaces or objects (fomite transmission) is also highly possible. SARS-CoV-2 can infect individuals of all ages; however, the elderly, individuals with weakened immunity, and those with underlying chronic pre-existing medical conditions such as chronic respiratory diseases, diabetes mellitus, hypertension, and cardiovascular diseases are more susceptible to contracting COVID-19 and more likely to experience serious illnesses due to COVID-19.

Since December 2019, the pandemic has evolved into several waves of infections linked to the emergence of various SARS-CoV-2 mutated variants known as the alpha (B.1.1.7), beta (B.1.351), delta (B.1.617.2) and omicron (B.1.1.529) variants. Comparisons of the variations in transmissibility, clinical characteristics, severity, and outcomes between patients infected with early and late variants were examined in a few previous studies. In particular, studies have shown that late variants are characterized by higher transmissibility (delta: 43–68% more transmissible than previously circulating variants [1], omicron: 36.5% more transmissible than delta [2]) compared to early variants (alpha and beta: 40–70% more transmissible compared to the original virus [3]). Although the transmissibility rate of COVID-19 late variants is considered to be higher than the original virus and early circulating variants, the severity of symptoms and clinical outcomes among variants has also changed slightly. For instance, the delta variant has been shown to be associated with more severe symptoms and about is twice as likely to require hospitalization than those infected with earlier circulating variants [4], whereas the omicron variant appears to be markedly less severe than early circulating variants [5] possibly due to the increased protection from COVID 19 vaccination status and cell-mediated immunity derived from previous infection. With regards to vaccination, the delta and omicron variants were predominant during the period of the widespread dissemination of COVID-19 vaccines, which began on mid-December 2020. Although the emergence of COVID-19 vaccines contributed to mitigate the severity and hospitalization posed by COVID-19, these COVID-19 variants are still considered very concerning due to the probable breakthrough infections.

SARS-CoV-2 variants are generally characterized by a number of clinical manifestations, although slight differences in the proportion of some symptoms between variants also emerged. Since the beginning of the COVID-19 pandemic, variable degrees ranging from mild to severe self-reported symptoms have been reported and generally include cough, fever, difficulty breathing, and some digestive symptoms including diarrhea and vomiting [6]. Furthermore, the acute phase of COVID-19 may also be accompanied by some peripheral and central neurological manifestations such as memory or cognitive disturbances, disturbed consciousness, myalgia, headache, malaise or fatigue, and insomnia [7,8,9]. Additionally, sensory disturbances including anosmia, hyposmia, ageusia, hypogeusia, and audiovestibular complaints including dizziness, vertigo, tinnitus, and hearing loss were commonly reported COVID-19 manifestations [10]. Along with the evolution of COVID-19 variants, the proportion of some self-reported symptoms has also changed slightly. Although taste and smell disturbances were considered chief and distinctive symptoms of COVID-19, these symptoms became less common during the omicron variant compared to delta and earlier variants [11,12]. With regard to audiovestibular symptoms, several systematic reviews [13,14,15], case reports/series [16,17,18,19,20], self-report survey studies [10,21,22,23,24,25] and a few clinical testing studies [26,27,28], there were increased reports of hearing loss, tinnitus, dizziness, and vertigo among COVID-19 infected individuals. Nevertheless, the majority of these studies were performed during the predominant periods of the original virus and early circulating variants of the COVID-19 pandemic.

Considering the higher transmissibility of late COVID-19 variants, there is a need for studies that examine potential changes in audiovestibular symptomatology with changes in SARS-CoV-2 mutations, while also comparing the findings against uninfected control considering seasonal variations. Therefore, we aimed to compare whether audiovestibular symptoms of COVID-19 with the late variants (delta and omicron) differed from that of early circulating variants (alpha and beta).

## 2. Materials and Methods

### 2.1. Participants

We recruited COVID-positive participants in the respective predominance periods of early variants (alpha and beta) from 5 April and 8 November 2020, the delta variant from 20 July to 25 August 2021, and the omicron variant from 10 February to 15 March 2022, and compared the findings with that of a group of uninfected controls. Data from the alpha/beta variants were part of a previously published project [10]. COVID-19 was verified using reverse transcription polymerase chain reaction (RT-PCR) for nasopharyngeal or oropharyngeal swabs and conducted at the central and regional laboratories of the Saudi Center for Disease Prevention and Control. Potential COVID-19 positive and uninfected negative control participants were approached through social media outlets (WhatsApp, Facebook, and Snapchat). To ensure wide dissemination of the questionnaire to potential participants, a snowballing sampling was also used for both COVID-19 positive and control groups. Prospective participants were encouraged to invite other COVID-19 positive participants who likely fulfill the criteria of either group.

We included participants if they met the following inclusion criteria; (1) those who had positive COVID-19 test results on RT-PCR testing for the COVID-19 groups; (2) 18 years of age or older; and (3) to agree to participate in the study by answering to a consent question at the beginning of the questionnaire. The uninfected control group consists of individuals with no history of positive COVID-19 test results on RT-PCR testing, nor have been suspected of contracting COVID-19. These un-infected control participants were not informed that audiovestibular symptoms or manifestations was the topic of the study, rather they were being surveyed regarding general health perceptions during the COVID-19 pandemic and winter season. Patients with a previous history of audiovestibular disorders in both COVID-19 and control groups were also allowed to participate in the study, as our goal was to assess any subtle changes in self-reported audiovestibular function due to COVID-19. Additionally, there is evidence that tinnitus was significantly exacerbated during the COVID-19 pandemic among individuals with pre-existing chronic tinnitus with and without positive COVID-19 test results [29]. This was addressed by including a question in the questionnaires distributed to both COVID-19 positive and negative participants that specifically asked whether pre-existing tinnitus and hearing loss deteriorated during COVID-19. Our study was approved by the institutional review board of the University of Hail (Protocol number: H-2020-v001).

### 2.2. Questionnaire Design and Instrumentation

We conducted a cross-sectional study using a questionnaire to compare the occurrence of audiovestibular symptoms during the acute phase of the disease among early and late COVID-19 variants. The questionnaire was developed and validated as described previously (Supplementary Materials for the questionnaire). Briefly, the questionnaire contained an introduction detailing the aim of the study followed by consent information to ensure participant confidentiality and anonymity. Following the introductory section, participants were required to complete three sections. Section 1 aimed to collect sociodemographic (age, gender, occupation), general health (of pre-existing chronic co-morbidities) and general COVID-19 symptoms (cough, fever, breathing difficulties, headache, loss of smell and taste, joint/back pain, diarrhea, chest pain, sore throat, and runny nose) following a positive COVID-19 test result. Antiviral drugs, such as hydroxychloroquine, used in the management plan for COVID-19 are known to have ototoxic effects. To assess whether these antiviral drugs have an effect on the manifestations of audiovestibular symptoms, we asked participants to report if they were given any antiviral drugs following positive COVID-19 test results. Section 2 was designed to examine the frequency of experiencing auditory symptoms following a positive COVID-19 test result. Specifically, using a 4-point scale (no = 0, mild = 1, moderate = 2, severe = 3), we asked participants if they developed new auditory symptoms. Furthermore, participants with pre-existing auditory symptoms were asked if their symptoms deteriorated after contracting COVID-19. Vestibular symptoms following COVID-19 positive test results were assessed in Section 3. Specifically, we asked participants on a 4-point scale (no = 0, mild = 1, moderate = 2, severe = 3) if they experienced vestibular symptoms including unsteadiness, dizziness, and vertigo. These three clinical terms may introduce confusion to participants and therefore participants were provided with the clinical term along with a brief description of each symptom. Participants were then asked if COVID-19 has exacerbated their vestibular symptoms if they had pre-existing chronic vestibular problems before COVID-19. Additionally, we also inquired about the duration over which they experienced auditory and/or vestibular symptoms. The questionnaire for the uninfected control group contained a mandatory question as to whether they ever tested positive for COVID-19. If the response was no, then they were prompted to proceed to questions related to the seasonal flu if they indicated they contracted the flu during the pandemic with the same wording as to those given the COVID-19 questionnaire.

Face and content validity of the questionnaire was determined by consulting two clinical audiologists. The audiologists were asked to independently provide a rating regarding the relevance, appropriateness, and clarity of each item in the questionnaire using a 4-point Likert scale; 1 = not relevant, 2 = somewhat relevant, 3 = relevant, 4 = very relevant. The responses from the reviewers indicated acceptable, clear, and relevant items of the questionnaire. Furthermore, the questionnaire was administered to four COVID-positive individuals for the evaluation of clarity of the questionnaire items on a 5-point scale, and question 26 was reported to be slightly ambiguous and it was reworded accordingly.

### 2.3. Data Analysis

Statistical analysis was conducted using the package for social sciences (SPSS) version 25.0 for Windows (SPSS Inc., Chicago, IL, USA). Descriptive statistical analyses were reported as percentages for categorical variables. Continuous variables were reported as mean and standard deviation for parametric variables or medians and interquartile ranges (IQRs) for non-parametric variables. For quantitative variables, we used the non-parametric Kruskal-Wallis test. Differences in auditory and vestibular symptoms between the four groups was examined using a Chi-square (x2) test. The Bonferroni adjustment method was used to compensate for multiple comparisons. Separate univariate and multivariate binary logistic regression analyses were conducted for symptom category (auditory and vestibular) to examine the relationship between audiovestibular symptoms and predictor variables (demographic, clinical characteristics and presentation, co-morbidities). Additionally, binary logistic regression analyses were also conducted to test the association between isolated auditory and vestibular symptoms and predictor variables (demographic, clinical characteristics and presentation, and co-morbidities). Odds ratio (OR), *p*-value (less than or equal to 0.05), and 95% confidence interval (CI) were used to identify any significant relationships between variables.

## 3. Results

### 3.1. Demographic and Clinical Characteristics

The total study sample included 939 participants comprising 120 uninfected controls and 794 COVID-19 positive patients including 301 that tested positive for COVID-19 when alpha/beta was dominant (between 5 April and 8 November 2020), 102 tested positive when the delta variant was dominant (between 20 July to 25 August 2021), and 416 when the omicron variant was dominant (between 10 February to 15 March 2022). Out of the 120 un-infected controls, 61 (50.83%) participants, surveyed when the omicron variant was dominant, reported that they contracted the seasonal flu. Table 1 shows the demographic characteristics and co-morbid medical conditions of un-infected participants and those with COVID-19 according to variant. The median age of the total study sample was 36 years (IQR, 29 to 43 years), with no statistically significant differences between groups (*p* = 0.580, Kruskal-Wallis test). The study population included 52.29% males with statistically significant differences in the distribution of gender between variants (*p* < 0.01), with more males when alpha/beta variants were dominant and more females when omicron variant was dominant. Overall, patients in the alpha/beta variant group (69%) had a higher rate of co-morbid medical conditions compared to un-infected controls (48%), delta (42%), and omicron (25%) variant groups (*p* < 0.01). The most frequent co-morbid medical conditions among COVID-19 infected individuals across variants were diabetes mellitus (8.9%), hypertension (8.9%), chronic respiratory diseases (6.4%), and anemia (5.9%).

### 3.2. COVID-19 Symptoms

**Figure 1:**
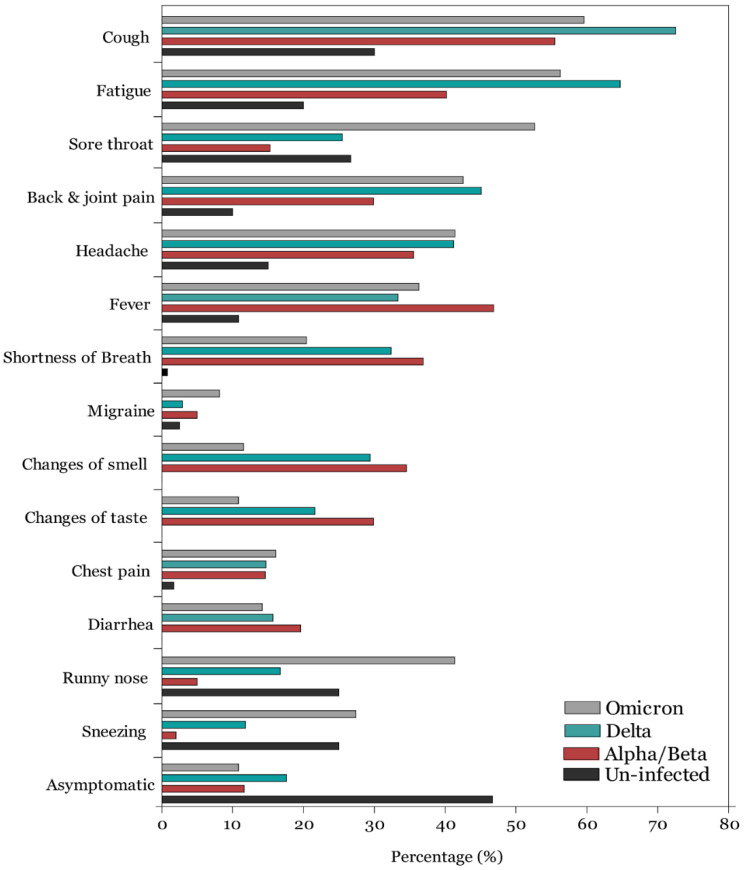
Frequency of COVID-19 symptoms among uninfected and infected participants when alpha/beta, delta, and omicron COVID-19 variants were dominant.

Overall, 10.82% (45/416) of COVID-19 infected individuals were asymptomatic in the omicron variant group compared to 17.65% (18/102) and 11.63% (35/301) in the delta and alpha/beta variants, respectively (Figure 1; Table S1 in Supplementary File) with no statistically significant differences among groups (*p* = 0.676). Among symptomatic individuals and those infected during the alpha/beta variant period, as reported in our previous study, the most frequently reported symptoms were cough, fever, fatigue, breathing difficulties, loss of smell, loss of taste, and back and joint pain. Among those infected during the delta variant, the most frequently reported symptoms were cough (72.54%), fatigue (64.71), headache (41.18%), back and joint pain (45.09%), fever (33.33%), breathing difficulties (32.35%), loss of smell (29.41%), and sore throat (25.49%). In contrast, among those infected with the omicron variant, the most frequently reported symptoms were cough (71.42%), fatigue (56.25%), sore throat (52.64), back and joint pain (42.55%), headache (41.38%), runny nose (41.34%), and sneezing (27.40%). Overall, loss of smell [OR = 0.25, 95% CI: 0.17–0.36)] and loss of taste [OR = 0.284, 95% CI: 0.19–0.42), *p* < 0.001] were significantly less prevalent during the omicron variant period compared to the alpha/beta and delta variants. In contrast, sore throat [OR = 6.16, 95% CI: 4.26–8.91, *p* < 0.001], runny nose [OR = 13.44, 95% CI: 7.72–23.40, *p* < 0.001], and sneezing [OR = 18.56, 95% CI: 8.04–42.84, *p* < 0.001] were significantly more prevalent during the omicron variant predominance period.

### 3.3. Auditory Symptoms

**Figure 2:**
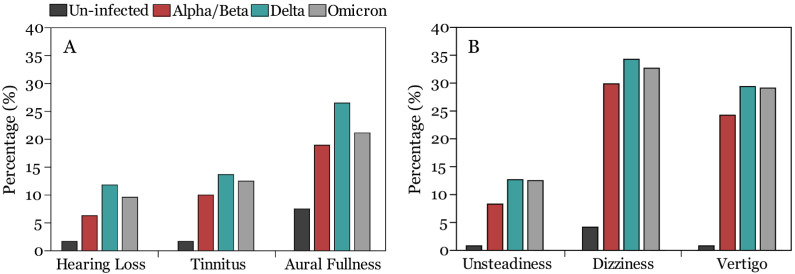
Frequency of auditory (**A**) and vestibular (**B**) symptoms among uninfected and infected participants when alpha/beta, delta, and omicron COVID-19 variants were dominant.

Analysis of the variation in audiovestibular symptoms occurrence across COVID-19 variants showed no qualitative and quantitative changes in the proportion of individuals reporting auditory symptoms (aural fullness, hearing loss, and tinnitus are combined as one entity) (Figure 2; Table S2 in Supplementary File). Among the COVID-19 variant groups, a total of 196 participants (23.9%) reported perceiving a form of auditory problems during the acute phase of infection compared to 11 (9.2%) uninfected controls (*p* < 0.001). Of note, the reports of auditory symptoms among un-infected controls were driven by those who contracted the seasonal flu. In binary logistic regression analysis, experiencing auditory symptoms was associated with loss of smell [OR = 1.73, 95% CI: 1.01–2.95, *p* = 0.004], chest pain [OR = 1.58, 95% CI: 1.00–2.49, *p* = 0.048], and back and joint pain [OR = 1.94, 95% CI: 1.34–2.80, *p* < 0.001].

Of the 196 COVID-19 positive participants who reported experiencing auditory symptoms, 73 (7.8%) reported hearing loss, 98 (10.4%) participants reported tinnitus, 181 (19.3%) participants reported aural fullness. In general, the proportion of individuals reporting auditory symptoms was slightly higher, although not statistically significant, among those infected during the delta variant (28.4%) compared to the alpha/beta (22.6%) and omicron (23.8%) variants’ predominance periods (Table 2).

A univariate binary logistic regression model showed those infected during the delta variant [OR = 2.89, 95% CI: 1.47–5.69, *p* = 0.002], delta [OR = 3.94, 95% CI: 1.85–8.37, *p* < 0.001], and omicron variant [OR = 3.09, 95% CI: 1.60–5.99, *p* = 0.001] predominance periods were more likely to experience hearing loss compared to uninfected controls, however, this turned out to be insignificant in multivariate logistic regression analysis (Table 3).

Nineteen (6.3%) participants that tested positive during the alpha/beta variants, 12 (11.8%) participants that tested positive during the delta variant, and 40 (9.6%) that tested positive during the omicron variants’ predominance period reported deterioration in hearing sensitivity during the acute phase of COVID-19 (Table 2). There was significant difference between the delta and omicron COVID-19 variant groups and the uninfected group (Table 2). However, there was no statistically significant difference between the three variants in self-reported changes in hearing sensitivity (*p* < 0.05, Chi-square test with Bonferroni correction) (Table 2). A binary logistic regression model revealed that reports of hearing difficulties was associated with gender [OR = 0.57, 95% CI: 0.33–0.99, *p* = 0.049], migraine [OR = 2.99, 95% CI: 1.33–6.63, *p* = 0.008], loss of smell [OR = 2.72, 95% CI: 1.25–6.63, *p* = 0.012], and back and joint pain [OR = 2.26, 95% CI: 1.26–4.04, *p* = 0.006] (Table S3 in Supplementary File).

Tinnitus during the acute stage of COVID-19 was reported by 30 (10%), 14 (13.7%), and 52 (12.5%) participants who tested positive during the alpha/beta, delta, and omicron variant predominance periods, respectively (Figure 2). There were significant differences between the three COVID-19 variant groups and the un-infected group (*p* < 0.05, Chi-square test with Bonferroni correction) (Table 2). However, there was no statistically significant difference among the three variants in self-reported experience of tinnitus (Table 2). In the binary logistic regression model, tinnitus was associated with the following predictors (age [OR = 1.03, 95% CI: 1.00–1.05, *p* = 0.029], migraine [OR = 2.37, 95% CI: 1.11–5.01, *p* = 0.024], and loss of smell [OR = 2.26, 95% CI: 1.12–4.59, *p* = 0.024], loss of taste [OR = 0.41, 95% CI: 0.19–0.89, *p* = 0.024], and back and joint pain [OR = 1.77, 95% CI: 1.07–2.95, *p* = 0.027]) (Table S5 in Supplementary File). Additionally, those infected during the omicron variant [OR = 4.56, 95% CI: 1.05–19.89, *p* = 0.043] predominance period were more likely to experience tinnitus compared to the alpha/beta [OR = 4.31, 95% CI: 0.92–20.22, *p* = 0.064] and delta variant [OR = 4.42, 95% CI: 0.91–21.61, *p* = 0.066] (Table S4 in Supplementary File).

In addition to lack of differences between variants in self-reports of hearing loss and tinnitus, we found that 57 (18.9%), 27 (26.5%), and 88 (21.2%) individuals reported experiencing aural fullness during the alpha/beta, delta, and omicron variants, respectively, compared to (7.5%) of the uninfected controls (Figure 2) with no significant differences between the variants (*p* < 0.05, Chi-square test with Bonferroni correction) (Table 2). Participants who experienced fatigue [OR = 1.58, 95% CI: 1.05–2.38, *p* = 0.029], loss of smell [OR = 1.77, 95% CI: 1.01–3.12, *p* = 0.047], and back and joint pain [OR = 1.78, 95% CI: 1.20–2.63, *p* = 0.004] were more likely to report aural fullness following a positive COVID-19 test (Table S5 in Supplementary File).

### 3.4. Vestibular Symptoms:

Overall, 289 (35.29%) members of the COVID-19 infected participants reported vestibular symptoms following positive COVID-19 test results compared to seven (5.8%) uninfected controls (*p* < 0.001) (Table 2 and Table S6 in Supplementary File). A binary regression analysis showed that experiencing vestibular symptoms was significantly associated with gender [OR = 1.48, 95% CI: 1.06–2.06, *p* = 0.02], shortness of breath [OR = 1.49, 95% CI: 1.05–2.15, *p* = 0.028], fatigue [OR = 1.83, 95% CI: 1.38–2.76, *p* = 0.001], and headache [OR = 1.57, 95% CI: 1.15–2.23, *p* = 0.005] (Table 4). Participants who tested positive during alpha/beta [OR = 5.58, 95% CI: 2.36–13.21, *p* < 0.001], delta [OR = 4.25, 95% CI: 1.69–10.65, *p* = 0.02], and omicron [OR = 4.12, 95% CI: 1.81–9.39, *p* = 0.001] variants were more likely to experience vestibular symptoms compared to uninfected controls (Table 4).

Of the 289 COVID-19 positive participants with vestibular symptoms, 261 (31.9%) participants reported dizziness, 216 (27.2%) participants reported vertigo, and 90 (11%) reported unsteadiness (Table 2). When compared across variants, unsteadiness was reported by 25 (8.3%), 13 (12.7%), and 52 (12.5%) participants in the alpha/beta, delta, and omicron variants, respectively, with no significant differences between variants (*p* < 0.05) (Table 2). Participants infected during alpha/beta [OR = 8.37, 95% CI: 1.04–67.56, *p* = 0.046], delta [OR = 8.69, 95% CI: 1.05–71.68, *p* = 0.045] and omicron [OR = 8.37, 95% CI: 1.11–63.35, *p* = 0.040] were more likely to experience unsteadiness following positive COVID-19 test results. Furthermore, unsteadiness was significantly associated with cough [OR = 1.81, 95% CI: 1.04–3.15, *p* = 0.036] and fatigue [OR = 2.07, 95% CI: 1.19–3.62, *p* = 0.001] as COVID-19 symptoms (Table S7 in Supplementary File).

Dizziness was reported by 90 (29.9%), 35 (34.3%), and 136 (29.1%) participants during the alpha/beta, delta, and omicron variants, with no significant differences between groups (*p* < 0.05) (Table 2). Participants who tested positive during the alpha/beta variant [OR = 7.39, 95% CI: 2.69–20.26, *p* < 0.001], delta variant [OR = 5.88, 95% CI: 2.06–16.81, *p* = 0.001], and omicron variant [OR = 4.77, 95% CI: 1.83–12.42, *p* = 0.014] were more likely to experience dizziness compared to uninfected controls. Experiencing dizziness following positive COVID-19 test results was significantly associated with anemia [OR = 1.89, 95% CI: 1.01–3.45, *p* = 0.046] and headache [OR = 1.56, 95% CI: 1.11–2.20, *p* = 0.011] (Table S8 in Supplementary File).

There were no statistically significant differences between the three COVID-19 variants (24.3% vs. 29.4% vs. 29.1%) in the frequency of reporting rotatory vertigo (*p* < 0.05) (Table 2), and all were significantly different compared to the un-infected group in which only one un-infected participant reported vertigo during the study period. Binary logistic regression showed that participants who tested positive during the alpha/beta variant [OR = 23.77, 95% CI: 3.14–180.17, *p* < 0.001], delta variant [OR = 21.76, 95% CI: 2.81–168.73, *p* = 0.003], and omicron variant [OR = 20.69, 95% CI: 2.81–152.61, *p* = 0.003] were more likely to experience vertigo relative to uninfected controls (Table S9 in Supplementary File). Experiencing vertigo following a positive COVID-19 test result was significantly associated with anemia [OR = 1.95, 95% CI: 1.02–3.74, *p* = 0.034], fatigue [OR = 2.00, 95% CI: 1.36–2.97, *p* = 0.043], and headache [OR = 1.76, 95% CI: 1.22–2.54, *p* = 0.003]. Furthermore, females were more likely to experience vertigo [OR = 1.63, 95% CI: 1.12–2.36, *p* = 0.010] (Table S9 in Supplementary File).

We also analyzed the data from un-infected controls while considering only those who self-reported contracting the seasonal flu and who were surveyed when the omicron variant was circulating. Specifically, the proportion of vestibular symptoms (unsteadiness, dizziness, and vertigo) and tinnitus, but not hearing loss and aural fullness, were significantly different between the un-infected controls and COVID-19 infected individuals in all analyzed variants (Table S10 in Supplementary File).

## 4. Discussion

COVID-19 variants have different transmission characteristics and clinical manifestations compared to the original coronavirus and early circulating variants. We conducted a comparative cross-sectional study to examine whether the evolving COVID-19 variants were associated with differences in reporting audiovestibular symptoms during the predominance periods for alpha/beta, delta, and omicron variants compared to non-COVID controls. Overall, our findings showed that COVID-19 infected individuals in all variant periods reported significantly more audiovestibular symptoms compared to non-COVID controls. However, our findings did not show significant changes in audiovestibular symptoms across COVID-19 variants. Furthermore, individuals infected when the delta variant was dominant reported audiovestibular symptoms at a higher rate, although not statistically significant, than other variants (See Table 2).

Overall, about a quarter (24.02%) of all COVID-19 participants in all variants combined reported experiencing auditory symptoms; most commonly aural fullness (21%) followed by tinnitus (11.7%), and lastly hearing loss (8.7%). There were no significant differences in reporting auditory symptoms during the acute phase of all COVID-19 variants. Hearing loss estimates reported in our study were consistent with an earlier systematic review, conducted at the beginning of the pandemic, reporting a prevalence of about 7.6% [14]. Additionally, several studies revealed elevated hearing thresholds at high audiometric frequencies among COVID-19 patients [20,26,27,30,31]. In contrast, other studies comparing COVID-19 positive individuals with mild to moderate illness with asymptomatic individuals did not show significant differences in audiometric thresholds at any audiometric frequency. Two studies also reported a possible subclinical auditory dysfunction evidenced by reduced OAEs amplitudes [26,28,30] in conjunction with a lack of elevated audiometric thresholds [28]. Furthermore, COVID-19-related sudden sensorineural hearing loss was also reported in many case reports since the beginning of this pandemic [16,17,18,20,32,33]. Overall, these findings suggest that auditory symptoms are common during the acute phase of COVID-19, and comprehensive audiological testing is required to further elucidate the pathogenesis of the underlying auditory dysfunction.

Tinnitus following positive COVID-19 test results was reported in a total of 94 (11.7%) participants, of which 10%, 13.7% and 12.5% were infected during the alpha/beta, delta, and omicron variants, respectively, and this was significantly higher than the non-COVID controls (1.7%). These estimates are consistent with previous studies reporting a prevalence of tinnitus ranging from 0.35% to as high as 35% [21,22,25,29,34,35]. The literature suggests that tinnitus is strongly associated with psychological disorders such as anxiety, stress, and depression [36,37,38]. These psychological disorders have been shown to be prevalent among infected and non-infected individuals during the COVID-19 pandemic [39,40] and among those with chronic tinnitus during the COVID-19 pandemic [41]. A previous study including non-COVID-19 participants with chronic tinnitus and COVID-19 infected individuals revealed that psychological disorders may possibly lead to the development of new tinnitus among COVID-19 infected individuals and the exacerbation of tinnitus during the COVID-19 pandemic among those with pre-existing tinnitus [29]. In the context of the COVID-19 pandemic, the onset of tinnitus can be in part explained by stressful and anxiety-related experiences of being diagnosed with COVID-19 or due to factors related to social isolation and lockdown in cases when lockdowns were implemented during the predominance periods of early variants. Regression analyses revealed that reports of experiencing tinnitus was associated with some neurological symptoms including changes of taste and smell and migraine.

The increased odds of reporting auditory symptoms among those who reported changes of smell and taste as well as migraine following positive COVID-19 test results are consistent with our earlier report when the early variants were dominant [10]. It has been postulated that the causative agent of COVID-19 possibly infiltrates the central nervous system via the blood–brain barrier [42] or via the olfactory pathway [43]. The olfactory pathway entry point may in part explain the increased odds of experiencing changes of smell commonly reported by COVID-19 infected individuals as well as the increased odds of experiencing auditory symptoms found in our study. The pathogenesis of the manifestation of auditory symptoms following a positive COVID-19 test remains unclear. COVID-19 can cause inflammatory, vascular insult through coagulopathy and secondary immune mediated dysfunction. The severity or multitude of general symptoms could reflect the severity of the condition and be more likely to affect auditory and vestibular systems. Furthermore, COVID-19-related auditory symptoms can possibly be explained by the evidence that the cellular receptor for the SARS-CoV-2 (angiotensin-converting enzyme 2 [ACE-2] receptors) was confirmed to be present in multiple areas along the auditory pathway including the eustachian tube, middle ear tissues, hair cells, spiral ganglion cells, and in the stria vascularis [44,45]. Furthermore, findings from the original virus also suggested the presence of SARS-CoV-2 particles in the brainstem, a site where the primary afferent innervation of cochleovestibular nerves arises [46]. Additionally, the predominant report of aural fullness in all analyzed COVID-19 variants can also be explained by the fact that ACE-2 was also predominantly present in the eustachian tube [44,45], indicating that that the eustachian tube is likely susceptible to SARS-CoV-2 infection and consequently leads to reports of aural fullness.

Vestibular symptoms among COVID-19 infected individuals were reported more frequently in all COVID-19 analyzed variants compared to auditory symptoms. Specifically, vestibular symptoms were reported by 34.9%, 36.3%, and 35.3% of individuals, respectively, when the alpha/beat, delta, and omicron variants were dominant. A previous study performed comprehensive vestibular examinations on COVID-19 positive individuals and revealed significant abnormalities in video head impulse testing (vHIT), cervical and ocular vestibular evoked myogenic potentials compared to non-COVID controls [31], suggesting possible COVID-19 effects on the vestibular system. Our analysis also showed that anemia was significantly associated with self-reports of dizziness and vertigo. Previous studies indicated that anemia is a risk factor associated with severe COVID-19 disease and severe inflammatory responses as well as more organ damage [47]. The exact mechanism of anemia leading to inner ear manifestations such as auditory or vestibular disorders is still unclear. However, it is reasonable to speculate that the underlying mechanism of anemia-induced vestibular symptoms is related to inflammatory, metabolic, and vaso-occlusion of the blood supply to the inner ear. The inner ear vasculature is sensitive and thus vulnerable to insults that disrupt blood flow to the inner ear [48], in addition to them being end organs supplied by labyrinthine arteries with no collateral circulation. This compromised circulation to the inner ear structures may lead to increased susceptibility to developing thrombosis or hypoxia and therefore leads to vestibular disorders among COVID-19 patients with anemia.

A logistic regression analysis in the current study also showed that female participants are more likely to report vestibular symptoms compared to males. Gender differences with regards to vestibular disorders were reported in the literature demonstrating a significant female predominance with respect to developing vestibular disorders [49,50] such as vestibular neuritis, Meniere’s disease, benign paroxysmal positional vertigo (BPPV) [51], vestibular migraine [52], and mal de debarquement syndrome [53]. These gender differences have been hypothesized to be related to the hormonal influence, however, the exact mechanism as to why women develop vestibular disorders more frequently than men remains unclear [50].

Although participants were provided with the operational definition and description of each vestibular symptom analyzed, there may still be some overlap between reports of experienced symptoms. Moreover, it is still unclear whether the reports of vestibular symptoms following COVID-19 are of a central or peripheral nature. It is difficult to clearly differentiate between the multitude of causes of vestibular problems and whether it is of central or peripheral vestibular origin for the onset of dizziness and vertigo following positive COVID-19 test solely on the basis of self-report questionnaire without further history taking and vestibular testing. For instance, logistic regression analyses of the current study revealed that individuals with shortness of breath as a COVID-19 symptom are more likely to experience vestibular problems. Shortness of breath might reflect severity of the condition and the possible involvement of different body systems. Hypoxia and coagulopathy-related vascular insults could cause both central and peripheral vestibular disorders. Moreover, vestibular symptoms were also associated with headache and fatigue as COVID-19 symptoms. Angiopathic, hypoxic or immune mediated insults to the brain might cause headache and vestibular center insults. Additionally, COVID-19-related stress could trigger migraines, which can cause both central and peripheral vestibular manifestations. With regard to fatigue, diffuse macroangiopathic thrombi or cardiomyositis associated with COVID-19 could cause cardiac related fatigue and possible vascular vestibular insult. Furthermore, COVID-19 could cause myositis [54], possibly indicating that fatigue might be associated with the weakness of postural control muscles [55]. Fatigue may also lead to recumbency and decreased mobility which subsequently could cause orthostatic dizziness/hypotension. Taken together, there is overlap between the origin of vestibular symptoms and disorders and, therefore, careful history taking in addition to physical and vestibular examinations is required to unravel the etiopathology of vestibular manifestations as a result of COVID-19.

The current study has some limitations. First, data concerning gene sequencing for the analyzed COVID-19 variants were not available to the researcher. However, personal communication with the Saudi surveillance center revealed that selected samples of positive RT-PCR tests of late variants accounted for the majority of cases when these variants were dominant. Second, the cross-sectional survey design of our study does not substitute comprehensive audiological and vestibular testing to elucidate the nature of auditory and vestibular dysfunction caused by COVID-19.

## 5. Conclusions

Our study demonstrates that audiovestibular symptoms are common during the acute phase of alpha/beta, delta, and omicron COVID-19 variants. These findings demonstrate the need for otolaryngologists and audiologists to perform physical, behavioral, and electrophysiological audiovestibular examinations to determine the possible etiopathology of audiovestibular dysfunction associated with COVID-19. Although the emergence and worldwide dissemination of COVID-19 vaccines may generally reduce the impact posed by COVID-19 variants, recognition and monitoring of audiovestibular symptoms as COVID-19 manifestations are still very important in the management of the disease. Future studies are required to determine if these audio-vestibular symptoms are persistent beyond the acute phase of COVID-19.

## Figures and Tables

**Table 1:** Demographic, co-morbidities, and clinical characteristics of un-infected (n = 120) and COVID-19 infected participants when alpha/beta (n = 301), delta (n = 101), and omicron (n = 416) variants were dominant.

	Un-Infected (N = 120)	Alpha/Beta (N = 301)	Delta Variant (N = 102)	Omicron Variant (N = 416)	*p*-Value
	N (%)	N (%)	N (%)	N (%)	
Age					
Median	34	35	36	36	0.380
Interquartile Range	(26, 42)	(27, 44)	(28.5, 44)	(30, 42)	
Sex					<0.001
Male	83 (69.17)	211 (70.10)	53 (52)	143 (34.38)	<0.001
Female	37 (30.83)	90 (29.90)	49 (48)	273 (65.63)	<0.001
Comorbidities					
No Comorbidities	83 (69.17)	207 (68.77)	47 (46.08)	312 (75)	<0.001
Diabetes mellitus	8 (6.76)	39 (12.96)	13 (12.75)	21 (5.05)	0.001
Hypertension	8 (6.67)	35 (11.63)	12 (11.76)	26 (6.25)	0.041
Heart diseases	1 (0.83)	6 (1.99)	5 (4.90)	1 (0.24)	0.003
Chronic respiratory diseases	4 (3.33)	14 (4.65)	9 (8.82)	30 (7.21)	0.064
Anemia	5 (4.17)	15 (4.98)	9 (8.82)	24 (5.77)	0.442
Head trauma	0	2 (0.66)	0	2 (0.48)	0.744
Antiviral drugs					<0.001
Hydroxychloroquine	-	55 (18.27)	4 (3.19)	4 (0.96)	<0.001
Favipiravir	-	40 (13.29)	4 (3.19)	1 (0.42)	<0.001
Remdesivir	-	3 (0.99)	1 (0.98)	2 (0.48)	>0.05
Dexamethasone	-	10 (3.32)	3 (2.94)	3 (0.72)	0.001

**Table 2:** Post-hoc analyses with Bonferroni correction for auditory and vestibular symptoms across variants compared to uninfected controls.

	Control	Alpha/Beta	Delta	Omicron	COVID-19 Total	Total
Auditory symptoms	11 (9.2%) _a_	68 (22.6%) _b_	29 (28.4%) _b_	99 (23.8%) _b_	196 (24.02%)	207 (22%)
Hearing Loss	2 (1.7%) _a_	19 (6.3%) _a,b_	12 (11.8%) _b_	40 (9.6%) _b_	71 (8.7%)	73 (7.8%)
Tinnitus	2 (1.7%) _a_	30 (10%) _b_	14 (13.7%) _b_	52 (12.5%) _b_	94 (11.7%)	98 (10.4%)
Aural Fullness	9 (7.5%) _a_	57 (18.9%) _b_	27 (26.5%) _b_	88 (21.2%) _b_	172 (21%)	181 (19.3%)
Vestibular symptoms	7 (5.8%) _a_	105 (34.9%) _b_	37 (36.3%) _b_	147 (35.3%) _b_	289 (35.3%)	296 (31.5%)
Unsteadiness	1 (0.8%) _a_	25 (8.3%) _b_	13 (12.7%) _b_	52 (12.5%) _b_	90 (11%)	91 (9.7%)
Dizziness	5 (4.2%) _a_	90 (29.9%) _b_	35 (34.3%) _b_	136 (29.1%) _b_	261 (31.9%)	266 (28.3%)
Vertigo	1 (0.8%) _a_	73 (24.3%) _b_	30 (29.4%) _b_	121 (29.1%) _b_	216 (27.2%)	224 (23.9%)

Same subscripts denote a subset of groups whose proportions were not significantly different from each other at the 0.05 level.

**Table 3:** Univariate and multivariate binary logistic regression for auditory symptoms (hearing loss, tinnitus, and aural fullness).

	Univariate Regression		Multivariate Regression	
	OR (95% CI)	*p*-Value	OR (95% CI)	*p*-Value
Group (R-Control)		0.004 *		0.415
Alpha/Beta	2.89 (1.47–5.69)	0.002 *	1.71 (0.83–3.53)	0.150
Delta	3.94 (1.85–8.37)	<0.001 *	1.98 (0.88–4.46)	0.097
Omicron	3.09 (1.60–5.99)	0.001 *	1.63 (0.81–3.28)	0.175
Gender (R-Male)	1.45 (1.7–1.98)	0.018 *	1.09 (0.77–1.56)	0.616
Age	1 (0.99–1.02)	0.970	-	-
Diabetes	0.86 (0.49–1.52)	0.303	-	-
Hypertension	0.93 (0.54–1.63)	0.810	-	-
Cardiovascular	1.58 (0.48–5.19)	0.449	-	-
Respiratory diseases	1.47 (0.82–2.64)	0.197	-	-
Anemia	1.89 (1.5–3.42)	0.034 *	1.43 (0.75–2.72)	0.274
Head injury	1.18 (0.12–11.4)	0.887	-	-
Cough	1.60 (1.16–2.21)	0.004 *	0.96 (0.66–1.38)	0.817
Shortness of breath	1.44 (1.02–2.02)	0.039 *	0.86 (0.58–1.29)	0.476
Fatigue	2.55 (1.85–3.52)	<0.001 *	1.39 (0.95–2.04)	0.094
Fever	1.93 (1.41–2.64)	<0.001 *	1.23 (0.85–1.77)	0.267
Headache	2.16 (1.58–2.96)	<0.001 *	1.36 (0.95–1.95)	0.099
Migraine	2.13 (1.20–378)	0.010 *	1.51 (0.82–2.79)	0.194
Loss of smell	2.33 (1.63–3.32)	<0.001 *	1.73 (1.01–2.95)	0.044 *
Loss of taste	1.86 (1.27–2.72)	0.001 *	0.86 (0.49–1.52)	0.611
Chest pain	2.46 (1.65–3.66)	<0.001 *	1.58 (1.00–2.49)	0.048 *
Diarrhea	1.63 (1.08–2.44)	0.019 *	0.96 (0.61–1.50)	0.842
Back and joint pain	3.11 (2.26–4.27)	<0.001 *	1.94 (1.34–2.80)	<0.001 *
Sore throat	1.37 (0.99–1.89)	0.051 *	-	-
Runny nose	1.26 (0.89–1.77)	0.191	-	-
Sneezing	1.25 (0.84–1.85)	0.271	-	-
Antiviral drugs	0.86 (0.67–1.09)	0.223	-	-

R: reference, OR: odds ratio, CI: confidence interval. Regression analyses for specific vestibular symptoms in isolation see Tables S4–S6 in Supplementary File. (* *p* < 0.05).

**Table 4:** Univariate and multivariate binary logistic regression for vestibular symptoms (unsteadiness, dizziness, and vertigo).

	Univariate Regression		Multivariate Regression	
	OR (95% CI)	*p*-Value	OR (95% CI)	*p*-Value
Group (R-Control)	1.31 (1.16–1.49)	<0.001 *	1.15 (0.98–1.35)	0.094
Alpha/Beta	8.65 (3.89–19.23)	<0.001 *	5.58 (2.36–13.21)	<0.001 *
Delta	9.22 (3.78–22.54)	<0.001 *	4.25 (1.69–10.65)	0.002 *
Omicron	8.82 (4.01–19.43)	<0.001 *	4.12 (1.81–9.39)	0.001 *
Gender (R-Male)	1.89 (1.43–2.51)	<0.001 *	1.48 (1.06–2.06)	0.020 *
Age	1.01 (0.99–1.02)	0.163	-	-
Diabetes	0.91 (0.51–1.49)	0.701	-	-
Hypertension	0.79 (0.48–1.32)	0.378	-	-
Cardiovascular	1.88 (0.63–5.64)	0.260	-	-
Respiratory	1.22 (0.70–2.11)	0.488	-	-
Anemia	2.03 (1.16–3.54)	0.013 *	1.56 (0.84–2.91)	0.158
Head injury	0.78 (0.08–6.98)	0.779	-	-
Cough	2.04 (1.53–2.72)	<0.001 *	1.09 (0.78–1.54)	0.605
Shortness of breath	2.45 (1.80–3.33)	<0.001 *	1.49 (1.05–2.15)	0.028 *
Fatigue	3.46(2.58–4.63)	<0.001 *	1.83 (1.38–2.76)	0.001 *
Fever	2.22 (1.67–2.95)	<0.001 *	1.92 (0.88–1.73)	0.215
Headache	2.82 (2.12–3.75)	<0.001 *	1.57 (1.15–2.23)	0.005 *
Migraine	1.89 (1.09–3.27)	0.024 *	1.22 (0.63–2.14)	0.640
Loss of smell	2.27 (1.63–3.16)	<0.001 *	1.53 (0.85–2.29)	0.192
Loss of taste	1.98 (1.39–2.81)	<0.001 *	0.88 (0.53–1.51)	0.675
Chest pain	2.4. (1.65–3.51)	<0.001 *	1.15 (0.68–1.62)	0.828
Diarrhea	1.75 (1.21–2.55)	0.003 *	0.96 (0.59–1.36)	0.606
Back and joint pain	2.81 (2.11–3.74)	<0.001 *	1.31 (0.97–1.92)	0.074
Sore throat	1.58 (1.188–2.09)	0.002 *	1.06 (0.77–1.57)	0.606
Runny nose	1.34 (0.99–1.82)	0.060	-	-
Sneezing	1.58 (1.12–2.45)	0.010 *	1.28 (0.84–1.99)	0.244
Antiviral drugs	1.08 (0.88–1.31)	0.444	-	-

R: reference, OR: odds ratio, CI: confidence interval. Regression analyses for specific vestibular symptoms in isolation see Tables S7–S9 in Supplementary File. (* *p* < 0.05).

## Data Availability

Not applicable.

## Supplementary Materials

The following supporting information can be downloaded at: https://www.mdpi.com/article/10.3390/audiolres12060065/s1, Table S1: Frequency of self-reported COVID-19 symptoms experienced by participants during the acute phase of infection, Table S2: Frequency of self-reported auditory symptoms among patients with COVID-19 during the acute phase of the disease, Table S3: Multivariate logistic binary regression analysis for hearing loss reported during the acute phase of COVID-19 disease, Table S4: Multivariate logistic binary regression analysis for tinnitus reported during the acute phase of COVID-19 disease, Table S5: Multivariate logistic binary regression analysis for aural fullness reported during the acute phase of COVID-19 disease, Table S6: Frequency of self-reported vestibular symptoms among patients with COVID-19 during the acute phase of the disease, Table S7: Multivariate logistic binary regression analysis for unsteadiness reported during the acute phase of COVID-19 disease, Table S8: Multivariate logistic binary regression analysis for dizziness reported during the acute phase of COVID-19 disease, Table S9: Multivariate logistic binary regression analysis for vertigo reported during the acute phase of COVID-19 disease, Table S10: Univariate logistic binary regression analysis for auditory and vestibular symptoms reported during the acute phase of COVID-19 variants while considering only un-infected controls who self-reported contracting the seasonal flu (reference group: uninfected controls with seasonal flu). Supplementary Materials: Questionnaire. Knowledge and Awareness of Cytomegalovirus among.

## References

[1] Campbell F., Archer B., Laurenson-Schafer H., Jinnai Y., Konings F., Batra N., Pavlin B., Vandemaele K., Van Kerkhove M.D., Jombart T. (2021). Increased transmissibility and global spread of SARS-CoV-2 variants of concern as at June 2021. Eurosurveillance.

[2] Yang W., Shaman J.L. (2022). COVID-19 pandemic dynamics in South Africa and epidemiological characteristics of three variants of concern (Beta, Delta, and Omicron). medRxiv.

[3] Graham M.S., Sudre C.H., May A., Antonelli M., Murray B., Varsavsky T., Kläser K., Canas L.S., Molteni E., Modat M. (2021). Changes in symptomatology, reinfection, and transmissibility associated with the SARS-CoV-2 variant B.1.1.7: An ecological study. Lancet Public Health.

[4] Twohig K.A., Nyberg T., Zaidi A., Thelwall S., Sinnathamby M.A., Aliabadi S., Seaman S.R., Harris R.J., Hope R., Lopez-Bernal J. (2022). Hospital admission and emergency care attendance risk for SARS-CoV-2 delta (B.1.617.2) compared with alpha (B.1.1.7) variants of concern: A cohort study. Lancet Infect. Dis..

[5] Nyberg T., Ferguson N.M., Nash S.G., Webster H.H., Flaxman S., Andrews N., Hinsley W., Bernal J.L., Kall M., Bhatt S. (2022). Comparative analysis of the risks of hospitalisation and death associated with SARS-CoV-2 omicron (B.1.1.529) and delta (B.1.617.2) variants in England: A cohort study. Lancet.

[6] Mehta O.P., Bhandari P., Raut A., Kacimi S.E.O., Huy N.T. (2020). Coronavirus Disease (COVID-19): Comprehensive Review of Clinical Presentation. Front. Public Health.

[7] Liguori C., Pierantozzi M., Spanetta M., Sarmati L., Cesta N., Iannetta M., Ora J., Mina G.G., Puxeddu E., Balbi O. (2020). Subjective neurological symptoms frequently occur in patients with SARS-CoV2 infection. Brain Behav. Immun..

[8] Mao L., Jin H., Wang M., Hu Y., Chen S., He Q., Chang J., Hong C., Zhou Y., Wang D. (2020). Neurologic Manifestations of Hospitalized Patients with Coronavirus Disease 2019 in Wuhan, China. JAMA Neurol..

[9] Li Z., Liu T., Yang N., Han D., Mi X., Li Y., Liu K., Vuylsteke A., Xiang H., Guo X. (2020). Neurological manifestations of patients with COVID-19: Potential routes of SARS-CoV-2 neuroinvasion from the periphery to the brain. Front. Med..

[10] Almishaal A.A., Alrushaidan A.A. (2022). Short- and Long-Term Self-Reported Audiovestibular Symptoms of SARS-CoV-2 Infection in Hospitalized and Nonhospitalized Patients. Audiol. Neurotol..

[11] Menni C., Valdes A.M., Polidori L., Antonelli M., Penamakuri S., Nogal A., Louca P., May A., Figueiredo J.C., Hu C. (2022). Symptom prevalence, duration, and risk of hospital admission in individuals infected with SARS-CoV-2 during periods of omicron and delta variant dominance: A prospective observational study from the ZOE COVID Study. Lancet.

[12] Vihta K.-D., Pouwels K.B., Peto T.E., Pritchard E., House T., Studley R., Rourke E., Cook D., Diamond L., Crook D. (2022). Omicron-associated changes in SARS-CoV-2 symptoms in the United Kingdom. Clin. Infect. Dis..

[13] Almufarrij I., Munro K.J. (2021). One year on: An updated systematic review of SARS-CoV-2, COVID-19 and audio-vestibular symptoms. Int. J. Audiol..

[14] AlMufarrij I., Uus K., Munro K.J. (2020). Does coronavirus affect the audio-vestibular system? A rapid systematic review. Int. J. Audiol..

[15] Bayat A., Saki N., Nikakhlagh S., Mirmomeni G., Raji H., Soleimani H., Rahim F. (2019). Is COPD associated with alterations in hearing? A systematic review and meta-analysis. Int. J. Chronic Obstr. Pulm. Dis..

[16] Degen C., Lenarz T., Willenborg K. (2020). Acute Profound Sensorineural Hearing Loss After COVID-19 Pneumonia. Mayo Clin. Proc..

[17] Koumpa F.S., Forde C.T., Manjaly J.G. (2020). Sudden irreversible hearing loss post COVID-19. BMJ Case Rep..

[18] Lamounier P., Franco V., Ramos H.V.L., Gobbo D.A., Teixeira R.P., Dos Reis P.C., Fayez B., Cândido C.C. (2020). A 67-Year-Old Woman with Sudden Hearing Loss Associated with SARS-CoV-2 Infection. Am. J. Case Rep..

[19] Lang B., Hintze J., Conlon B. (2020). Coronavirus disease 2019 and sudden sensorineural hearing loss. J. Laryngol. Otol..

[20] Ricciardiello F., Pisani D., Viola P., Cristiano E., Scarpa A., Giannone A., Longo G., Russo G., Bocchetti M., Coppola C. (2021). Sudden Sensorineural Hearing Loss in Mild COVID-19: Case Series and Analysis of the Literature. Audiol. Res..

[21] AlJasser A., Alkeridy W., Munro K.J., Plack C.J. (2021). Is COVID-19 associated with self-reported audio-vestibular symptoms?. Int. J. Audiol..

[22] Micarelli A., Granito I., Carlino P., Micarelli B., Alessandrini M. (2020). Self-perceived general and ear-nose-throat symptoms related to the COVID-19 outbreak: A survey study during quarantine in Italy. J. Int. Med. Res..

[23] Munro K.J., Uus K., AlMufarrij I., Chaudhuri N., Yioe V. (2020). Persistent self-reported changes in hearing and tinnitus in post-hospitalisation COVID-19 cases. Int. J. Audiol..

[24] Savtale S., Hippargekar P., Bhise S., Kothule S. (2021). Prevalence of Otorhinolaryngological Symptoms in Covid 19 Patients. Indian J. Otolaryngol. Head Neck Surg..

[25] Viola P., Ralli M., Pisani D., Malanga D., Sculco D., Messina L., Laria C., Aragona T., Leopardi G., Ursini F. (2020). Tinnitus and equilibrium disorders in COVID-19 patients: Preliminary results. Eur. Arch. Otorhinolaryngol..

[26] Mustafa M. (2020). Audiological profile of asymptomatic COVID-19 PCR-positive cases. Am. J. Otolaryngol..

[27] de Sousa F.A., Costa R.P., Xará S., Pinto A.N., e Sousa C.A. (2021). SARS-CoV-2 and hearing: An audiometric analysis of COVID-19 hospitalized patients. J. Otol..

[28] Daikhes N., Карнеева O., Machalov A., Kuznetcov A., Sapozhnikov Y., Balakina A., Khulugurova L., Карпов V. (2020). Audiological profile of patients with SARS-Co-V-2 PCR-positive cases. Vestnik Otorinolaringol..

[29] Beukes E.W., Baguley D.M., Jacquemin L., Lourenco M.P.C.G., Allen P.M., Onozuka J., Stockdale D., Kaldo V., Andersson G., Manchaiah V. (2020). Changes in Tinnitus Experiences During the COVID-19 Pandemic. Front. Public Health.

[30] Öztürk B., Kavruk H., Aykul A. (2022). Audiological findings in individuals diagnosed with COVID-19. Am. J. Otolaryngol..

[31] Tan M., Cengiz D.U., Demir I., Demirel S., Çolak S.C., Karakaş O., Bayındır T. (2022). Effects of Covid-19 on the audio-vestibular system. Am. J. Otolaryngol..

[32] Kilic O., Kalcioglu M.T., Cag Y., Tuysuz O., Pektas E., Caskurlu H., Cetın F. (2020). Could sudden sensorineural hearing loss be the sole manifestation of COVID-19? An investigation into SARS-CoV-2 in the etiology of sudden sensorineural hearing loss. Int. J. Infect. Dis..

[33] Meng X., Wang J., Sun J., Zhu K. (2022). COVID-19 and Sudden Sensorineural Hearing Loss: A Systematic Review. Front. Neurol..

[34] Davis H.E., Assaf G.S., McCorkell L., Wei H., Low R.J., Re’Em Y., Redfield S., Austin J.P., Akrami A. (2020). Characterizing long COVID in an international cohort: 7 months of symptoms and their impact. medRxiv.

[35] Lechien J.R., Chiesa-Estomba C.M., De Siati D.R., Horoi M., Le Bon S.D., Rodriguez A., Dequanter D., Blecic S., El Afia F., Distinguin L. (2020). Olfactory and gustatory dysfunctions as a clinical presentation of mild-to-moderate forms of the coronavirus disease (COVID-19): A multicenter European study. Eur. Arch. Otorhinolaryngol..

[36] Zöger S., Svedlund J., Holgers K.-M. (2006). Relationship Between Tinnitus Severity and Psychiatric Disorders. J. Psychosom. Res..

[37] Falkenberg E.-S., Wie O.B. (2012). Anxiety and Depression in Tinnitus Patients: 5-Year Follow-Up Assessment after Completion of Habituation Therapy. Int. J. Otolaryngol..

[38] Krog N.H., Engdahl B., Tambs K. (2010). The association between tinnitus and mental health in a general population sample: Results from the HUNT Study. J. Psychosom. Res..

[39] Passos L., Prazeres F., Teixeira A., Martins C. (2020). Impact on Mental Health Due to COVID-19 Pandemic: Cross-Sectional Study in Portugal and Brazil. Int. J. Environ. Res. Public Health.

[40] Salari N., Hosseinian-Far A., Jalali R., Vaisi-Raygani A., Rasoulpoor S., Mohammadi M., Rasoulpoor S., Khaledi-Paveh B. (2020). Prevalence of stress, anxiety, depression among the general population during the COVID-19 pandemic: A systematic review and meta-analysis. Glob. Health.

[41] Xia L., He G., Feng Y., Yu X., Zhao X., Yin S., Chen Z., Wang J., Fan J., Dong C. (2021). COVID-19 associated anxiety enhances tinnitus. PLoS ONE.

[42] Lima M., Siokas V., Aloizou A.-M., Liampas I., Mentis A.-F.A., Tsouris Z., Papadimitriou A., Mitsias P.D., Tsatsakis A., Bogdanos D.P. (2020). Unraveling the Possible Routes of SARS-COV-2 Invasion into the Central Nervous System. Curr. Treat. Options Neurol..

[43] Giacomelli A., Pezzati L., Conti F., Bernacchia D., Siano M., Oreni L., Rusconi S., Gervasoni C., Ridolfo A.L., Rizzardini G. (2020). Self-reported Olfactory and Taste Disorders in Patients with Severe Acute Respiratory Coronavirus 2 Infection: A Cross-sectional Study. Clin. Infect. Dis..

[44] Frazier K.M., Hooper J.E., Mostafa H.H., Stewart C.M. (2020). SARS-CoV-2 Virus Isolated from the Mastoid and Middle Ear: Implications for COVID-19 Precautions During Ear Surgery. JAMA Otolaryngol. Head Neck Surg..

[45] Uranaka T., Kashio A., Ueha R., Sato T., Bing H., Ying G., Kinoshita M., Kondo K., Yamasoba T. (2020). Expression of ACE2, TMPRSS2, and Furin in Mouse Ear Tissue, and the Implications for SARS-CoV-2 Infection. Laryngoscope.

[46] Meinhardt J., Radke J., Dittmayer C., Franz J., Thomas C., Mothes R., Laue M., Schneider J., Brünink S., Greuel S. (2021). Olfactory transmucosal SARS-CoV-2 invasion as a port of central nervous system entry in individuals with COVID-19. Nat. Neurosci..

[47] Tao Z., Xu J., Chen W., Yang Z., Xu X., Liu L., Chen R., Xie J., Liu M., Wu J. (2021). Anemia is associated with severe illness in COVID-19: A retrospective cohort study. J. Med. Virol..

[48] Trune D.R., Nguyen-Huynh A. (2012). Vascular Pathophysiology in Hearing Disorders. Semin. Heart.

[49] Zanon A., Sorrentino F., Franz L., Brotto D. (2019). Gender-related hearing, balance and speech disorders: A review. Heart Balance Commun..

[50] Smith P.F., Agrawal Y., Darlington C.L. (2019). Sexual dimorphism in vestibular function and dysfunction. J. Neurophysiol..

[51] Hülse R., Biesdorf A., Hörmann K., Stuck B., Erhart M., Hülse M., Wenzel A. (2019). Peripheral Vestibular Disorders: An Epidemiologic Survey in 70 Million Individuals. Otol. Neurotol..

[52] Formeister E.J., Rizk H.G., Kohn M.A., Sharon J.D. (2018). The Epidemiology of Vestibular Migraine: A Population-based Survey Study. Otol. Neurotol..

[53] Cha Y.-H., Cui Y.Y., Baloh R.W. (2018). Comprehensive Clinical Profile of Mal De Debarquement Syndrome. Front. Neurol..

[54] Saud A., Naveen R., Aggarwal R., Gupta L. (2021). COVID-19 and Myositis: What We Know So Far. Curr. Rheumatol. Rep..

[55] Helbostad J.L., Sturnieks D.L., Menant J., Delbaere K., Lord S.R., Pijnappels M. (2010). Consequences of lower extremity and trunk muscle fatigue on balance and functional tasks in older people: A systematic literature review. BMC Geriatr..

