# Supplementary material for: Comparative Study of Audiovestibular Symptoms between Early and Late Variants of COVID-19
Source: Audiol Res. 2022 Dec 4;12(6):680–95. doi: 10.3390/audiolres12060065 (PMC9774134; doi:10.3390/audiolres12060065)
Supplement: Supplementary file 1 [file audiolres-12-00065-s001.zip › Audiovestibular Questionnaire_COVID.pdf]

Supplementary Materials: Questionnaire

Audio-vestibular manifestations among COVID 19 patients

A- Personal and clinical characteristics

أ- البيانات الشخصية

1- What is your age? ______ Years

١- العمر: ____ سنة

2- Gender:

☐ Male
☐ Female

٢- الجنس:

☐ ذكر
☐ أنثى

3- Occupation?

☐ Healthcare practitioner
☐ Teacher
☐ Governmental sector
☐ Private sector
☐ Military
☐ Unemployed
☐ Retired
☐ Other: ....

٣- ماهي وظيفتك؟

☐ موظف في المجال الصحي
☐ معلم
☐ موظف في القطاع الحكومي
☐ موظف في القطاع الخاص
☐ موظف عسكري
☐ غير موظف
☐ متقاعد
☐ أخرى: ....

4- Do you have any of the following chronic conditions?

☐ I don't have chronic diseases
☐ Diabetes
☐ Hypertension
☐ Cardiovascular diseases
☐ Immunosuppression
☐ Anemia
☐ Stroke
☐ Temporomandibular joint
☐ Head injury
☐ Other: ....

٤- هل لديك أي من الأمراض المزمنة التالية (يمكنك الإختيار أكثر من إجابة)؟

☐ ليس لدي أي أمراض مزمنة
☐ سكري
☐ ارتفاع ضغط الدم
☐ أمراض القلب
☐ أمراض تنفسية مزمنة (الربو – حساسية مزمنة)
☐ نقص المناعة
☐ أنيميا منجلية
☐ السكتة الدماغية
☐ اضطراب المفصل الصدغي الفكي
☐ إصابة بالرأس
☐ أخرى (أذكرها): ....

5- Did you experience any symptoms after contracting COVID 19?

☐ Yes
☐ No

٥- هل ظهرت لديك أي أعراض بعد إصابتك بفيروس كورونا؟

☐ نعم
☐ لا

6- If yes, what are these symptoms?

☐ Cough
☐ Breathing difficulties
☐ Fatigue and body weakness
☐ Fever
☐ Headache
☐ Migraine
☐ Loss of smell
☐ Loss of taste
☐ Chest pain
☐ Diarrhea
☐ Back and joints pain
☐ no symptoms
☐ Other: ....

٦- ما هي هذه الأعراض (يمكنك الإختيار أكثر من إجابة)؟

☐ كحة
☐ ضيق وصعوبات في التنفس
☐ خمول عام في الجسم
☐ ارتفاع درجة الحرارة
☐ صداع عام
☐ صداع نصفي
☐ فقدان حاسة الشم
☐ فقدان حاسة التذوق
☐ ألم في الصدر
☐ سهال
☐ ألم في المفاصل والظهر
☐ لا يوجد أعراض
☐ أخرى (أذكرها): ....

Supplementary Materials: Questionnaire

Audio-vestibular manifestations among COVID 19 patients

7- Were you admitted to hospital after contracting COVID 19?
- ☐ Yes
- ☐ No, home quarantine.

8- Were you admitted to the ICU after contracting COVID 19?
- ☐ Yes
- ☐ No

9- Mechanical ventilation?
- ☐ Yes
- ☐ No

10- Did you take any of the following antiviral drugs?
- ☐ Hydroxychloroquine
- ☐ Favipiravir
- ☐ Remdisivir
- ☐ Dixamethasone (anti-inflammatory)
- ☐ No

11- Please indicate any other medications such as pain killers you took (Panadol, Tylenol, Ibuprofen … etc)?

……………………………………………………………………

B- Auditory symptoms after contracting COVID 19

12-Do you have hearing or ear-related problems before contracting COVID 19?
- ☐ Yes, mild hearing loss
- ☐ Yes, moderate hearing loss
- ☐ Yes, severe hearing loss
- ☐ No

13-Do you have tinnitus before contracting COVID 19?
- ☐ Yes, intermittent
- ☐ Yes, continuous
- ☐ No

٧- هل تم تنويمك في المستشفى نتيجة إصابتك بفيروس كورونا؟
- ☐ نعم
- ☐ لا، العزل الطبي المنزلي

٨- هل أدخلت وحدة العناية المركزة من أعراض فيروس كورونا؟
- ☐ نعم
- ☐ لا

٩- هل تم وضع جهاز التنفس الصناعي عليك نتيجة إصابتك بفيروس كورونا؟
- ☐ نعم
- ☐ لا

١٠- هل تم إعطاؤك أدوية مضادة للفيروسات للتخفيف من أعراض فيروس كورونا؟
- ☐ لا
- ☐ نعم ، هيدروكسي كلوركوين
- ☐ نعم ،رامديسيفير
- ☐ نعم ، ديكساميثازون
- ☐ نعم ، فافيبيرافير

١١- أذكر أية أدوية أخرى قمت باستخدامها في فترة التشافي من فيروس كورونا (بنادول، أدول، الإيبوبروفين، ، الأسبيرين، تايلينول .. أخرى)؟

……………………………………………………………………

ب: الأعراض المتعلقة بمشاكل السمع نتيجة الإصابة بفيروس كورونا:

١٢- هل لديك ضعف سمع أو مشاكل متعلقة بالأذن <u>قبل إصابتك بفيروس كورونا</u>؟
- ☐ نعم ، ضعف سمع بسيط
- ☐ نعم ، ضعف سمع متوسط
- ☐ نعم ، ضعف سمع شديد
- ☐ لا

١٣- هل لديك طنين <u>قبل إصابتك بفيروس كورونا</u>؟
- ☐ نعم، طنين متقطع
- ☐ نعم، طنين مستمر
- ☐ لا

Supplementary Materials: Questionnaire

Audio-vestibular manifestations among COVID 19 patients

14- <u>After contracting COVID 19</u>, did you experience hearing loss?

☐ Yes, mild hearing loss
☐ Yes, moderate hearing loss
☐ Yes, severe hearing loss
☐ No

15- <u>After contracting COVID 19</u>, did you experience tinnitus (ringing or buzzing in your ears or head in the absence of external sounds)?

☐ Yes, mild tinnitus
☐ Yes, moderate tinnitus
☐ Yes, severe tinnitus
☐ No

16- <u>After contracting COVID 19</u>, did you experience aural fullness in your ears (blockage or ear pressure)?

☐ Yes, mild
☐ Yes, moderate
☐ Yes, severe
☐ No

17- <u>After contracting COVID 19</u>, did you experience speech understanding difficulties in quiet?

☐ Yes, mild
☐ Yes, moderate
☐ Yes, severe
☐ No

18- <u>After contracting COVID 19</u>, did you experience speech understanding difficulties in noise?

☐ Yes, mild
☐ Yes, moderate
☐ Yes, severe
☐ No

19- Please indicate the duration in which you experienced auditory symptoms?

………………………………………………………………

١٤- <u>بعد إصابتك بفيروس كورونا</u> هل ظهرت لديك أعراض ضعف سمع أو مشاكل في السمع؟

☐ نعم ، ضعف سمع بسيط
☐ نعم ، ضعف سمع متوسط
☐ نعم ، ضعف سمع شديد
☐ لا

١٥- <u>بعد إصابتك بفيروس كورونا</u> هل ظهر لديك طنين في الأذن (طنين الأذن هو مصطلح لسماع صوت في الرأس و / أو الأذنين في غياب أي صوت خارجي ، يستمر لأكثر من خمس دقائق في المرة الواحدة)؟

☐ نعم ، طنين بسيط
☐ نعم ، طنين متوسط
☐ نعم ، طنين شديد
☐ لا

١٦- <u>بعد إصابتك بفيروس كورونا</u> هل ظهر لديك إحساس بضغط او انسداد في الأذن؟

☐ نعم ، بسيط
☐ نعم ، متوسط
☐ نعم ، شديد
☐ لا

١٧- <u>بعد إصابتك بفيروس كورونا</u> هل ظهرت لديك مشاكل وصعوبة في فهم كلام الآخرين؟

☐ نعم، صعوبة بسيطة
☐ نعم، صعوبة متوسطة
☐ نعم، صعوبة شديدة
☐ لا، لم يكن لدي مشاكل في فهم كلام الآخرين بعد إصابتي بفيروس كورونا

١٨- <u>بعد إصابتك بفيروس كورونا</u> هل ظهرت لديك مشاكل في فهم كلام الآخرين خصوصاً في وجود الضجيج أو الضوضاء أو الإزعاج (مثال: في وجود مجموعة من المتحدثين – في مطعم ملئ بالإزعاج – في فهم كلام الآخرين أثناء متابعة التلفزيون)؟

☐ نعم، صعوبة بسيطة
☐ نعم، صعوبة متوسطة
☐ نعم، صعوبة شديدة
☐ لا

١٩- ما هي المدة التي استمرت فيها أعراض السمع (ضعف سمع - طنين الخ) ان كنت قد عانيت من تلك الأعراض؟ (الرجاء كتابة المدة. مثال: خمسة أيام)

………………………………………………………………

Supplementary Materials: Questionnaire

Audio-vestibular manifestations among COVID 19 patients

20- Did you have auditory symptoms and became worse after contracting COVID 19?

☐ Yes, tinnitus became worse

☐ Yes, hearing loss became worse

☐ Yes, I had auditory symptoms before COVID but did not became worse

☐ No, I did not have auditory problems before COVID 19

C- Vestibular symptoms after contracting COVID 19

21- <u>Before contracting COVID-19</u>, do you have vestibular problems?

☐ Yes, dizziness

☐ Yes, vertigo

☐ Yes, unsteadiness

☐ No

22- <u>After contracting COVID 19</u>, did you experience unsteadiness?

☐ Yes, mild

☐ Yes, moderate

☐ Yes, severe

☐ No

23-<u>After contracting COVID 19</u>, did you experience dizziness?

☐ Yes, mild

☐ Yes, moderate

☐ Yes, severe

☐ No

24- <u>After contracting COVID 19</u>, did you experience vertigo?

☐ Yes, mild

☐ Yes, moderate

☐ Yes, severe

☐ No

25- Did you have vestibular problems before COVID and became worse after contracting COVID 19?

☐ Yes, dizziness became worse

☐ Yes, vertigo became worse

☐ No, I had vestibular problems before COVID but did not became worse

☐ No, I did not have vestibular problems before COVID 19

٢٠- هل كنت تعاني بمشاكل في السمع قبل إصابتك بفيروس كورونا وأصبحت هذه الأعراض في حالة أسوء بعد إصابتك بفيروس كورونا؟

☐ نعم، ازدادت حدة الطنين

☐ نعم، مشاكل السمع أسوء بعد إصابتي بفيروس كورونا

☐ لا، كان لدي مشاكل في السمع والطنين قبل الإصابة بكورونا لكن لم تزداد سوءاً بعد الإصابة بالفيروس

☐ لا، لم يكن لدي ضعف سمع وطنين قبل الإصابة بكورونا

ج: الأعراض المتعلقة بمشاكل التوازن نتيجة الإصابة بفيروس كورونا

٢١- <u>قبل إصابتك بفيروس كورونا</u>، هل لديك مشاكل في التوازن مثل الدوران ودوخة؟

☐ نعم ، دوخة

☐ نعم ، دوران

☐ نعم مشاكل عامة في التوازن

☐ لا

٢٢- <u>بعد إصابتك بفيروس كورونا</u>، هل ظهرت لديك اضطرابات في التوازن(الشعور بعدم الاتزان او على على وشك فقدان التوازن).

☐ نعم، مشاكل في التوازن بسيطة

☐ نعم، مشاكل متوسطة الشدة في التوازن

☐ نعم، مشاكل شديدة في التوازن

☐ لا، لم تظهر لدي أية مشاكل في التوازن

٢٣- <u>بعد إصابتك بفيروس كورونا</u>، هل ظهرت لديك دوخة (عدم الثبات والاتزان أثناء الجلوس أو الوقوف أو المشي)؟

☐ نعم، دوخة بسيطة

☐ نعم، دوخة متوسطة الشدة

☐ نعم، دوخة شديدة

☐ لا

٢٤- <u>بعد إصابتك بفيروس كورونا</u>، هل ظهرت لديك احساس بالدوران (الشعور الزائف بالحركة أو الدوران سواءاً مان ذاتيا او الأشياء المحيطة (دوار)؟

☐ نعم، دوخة بسيطة

☐ نعم، دوخة متوسطة الشدة

☐ نعم، دوخة شديدة

☐ لا

٢٥- هل كنت تعاني بمشاكل في التوازن قبل إصابتك بفيروس كورونا وأصبحت هذه الأعراض في حالة أسوء بعد إصابتك بفيروس كورونا؟

☐ نعم، ازدادت شدة الدوخة

☐ نعم، ازدادت شدة الدوران

☐ لا، كان لدي مشاكل توازن قبل الإصابة بكورونا ولكن لم تزداد سوءاً

☐ لا، لم يكن لدي مشاكل في التوازن قبل إصابتي بفيروس كورونا

Supplementary Materials: Questionnaire

Audio-vestibular manifestations among COVID 19 patients

26- Please indicate the nature of your vestibular problems you experienced during COVID 19?

☐ Feeling of dizziness and vertigo while changing the position of the head, such as turning on the bed
☐ Dizziness with rapid head movement
☐ Dizziness accompanied by feeling of faint
☐ Dizziness and vertigo accompanied by headache as well as sensitivity to light and sound
☐ An episode of dizziness or spinning that lasts for several seconds
☐ An episode of dizziness and dizziness that lasts for days
☐ An episode of severe dizziness and vertigo that lasted for days accompanied by nausea and vomiting
☐ An episode of severe dizziness and vertigo that lasted from minutes to hours
☐ Feeling of dizziness and vertigo from visual stimuli (in the car while passing cars - in the supermarket - browsing a book or computer)

27- When did you start experiencing auditory and/or vestibular problems?

☐ First week of positive COVID 19 test
☐ Second week of positive COVID 19 test
☐ Third week of positive COVID 19 test
☐ I did not experience auditory or vestibular problems

28- Do you have any further comments?

....
....
....

Additional questions for individuals infected during the predominance periods of SARS-CoV-2 variants:

29- Have you had COVID before Delta or Omicron?
30- Did you take COVID-19vaccine?

٢٦- أختر من الخيارات التالية طبيعة مشاكل التوازن مثل الدوران ودوخة التي عانيتها بعد إصابتك بفيروس كورونا (اختر جميع ما ينطبق)؟

☐ لم أعاني من مشاكل في التوازن
☐ الإحساس بالدوار ودوخة أثناء تغيير وضع الجسم والرأس كالإنقلاب على السرير
☐ دوخة تأتي مع حركة الرأس السريعة
☐ دوخة ويرافقها الشعور بالإغماء
☐ الإصابة بالصداع مع الدوار والدوخة وكذلك حساسية من الضوء والضوضاء.
☐ نوبة دوخة أو الدوران تستمر لثواني عديدة
☐ نوبة دوخة ودوران شديدة استمرت لأيام
☐ نوبة دوخة ودوران شديدة استمرت لأيام ورافقها غثيان وقئ (استفراغ)
☐ نوبة دوخة ودوران شديدة استمرت من دقائق لساعات
☐ الشعور بالدوار والدوخة من المحفزات البصرية (في السيارة اثناء مرور السيارات – في السوبرماركت – في تصفح الكتاب او الكمبيوتر)

٢٧- متى بدأ ظهور أي أعراض متعلقة بالسمع والتوازن؟

☐ لم تظهر لدي أعراض متعلقة بالسمع والتوازن
☐ خلال الأسبوع الأول من ظهور نتائج فحص كورونا
☐ بعد الأسبوع الثاني من ظهور نتائج فحص كورونا
☐ بعد الأسبوع الثالث من ظهور نتائج فحص كورونا
☐ بعد الأسبوع الرابع من ظهور نتائج فحص كورونا
☐ بعد الأسبوع الخامس من ظهور نتائج فحص كورونا

٢٨- هل لديك أية تعليقات أو ملاحظات أخرى لم تذكر في الإستبيان؟

....
....
....

أسئلة إضافية للأشخاص المصابين أثناء ظهور متحور دلتا ومتحور أوميكرون:

٢٩- هل أصبت بكورونا قبل ظهور وانتشار أوميكرون ؟

٣٠- هل أخذت لقاح الوقاية من كورونا؟
