# Supplementary material for: Comparative Study of Audiovestibular Symptoms between Early and Late Variants of COVID-19
Source: Audiol Res. 2022 Dec 4;12(6):680–95. doi: 10.3390/audiolres12060065 (PMC9774134; doi:10.3390/audiolres12060065)
Supplement: Supplementary file 1 [file audiolres-12-00065-s001.zip › Supplementary Tables.pdf]

Table S1: Frequency of self-reported COVID-19 symptoms experienced by participants during the acute phase of infection.

**COVID-19 Symptoms**	**Un-infected (N=120) N (%)**	**Alpha & Beta (N=301) N (%)**	**Delta variant (N=102) N (%)**	**Omicron variant (N=416) N (%)**	***P*-value**
Asymptomatic	56 (46.67)	35 (11.63)	18 (17.6)	45 (10.82)	<0.001
Cough	36 (30)	167 (55.48)	74 (72.54)	248 (59.61)	<0.001
Fever	13 (10.83)	141 (46.84)	34 (33.33)	151 (36.29)	<0.001
Breathing difficulties	1 (0.8)	111 (36.88)	33 (32.35)	85 (20.43)	<0.001
Fatigue	24 (20)	121 (40.19)	66 (64.71)	234 (56.25)	<0.001
Headache	18 (15)	107 (35.55)	42 (41.18)	174 (41.83)	<0.001
Migraine	3 (2.5)	15 (4.98)	3 (2.9)	34 (8.17)	0.065
Loss of smell	0	104 (34.55)	30 (29.41)	48 (11.54)	<0.001
Loss of taste	0	90 (29.90)	22 (21.6)	45 (10.82)	<0.001
Chest pain	2 (1.67)	44 (14.62)	15 (14.7)	67 (16.11)	0.001
Diarrhea	0	59 (19.60)	16 (15.7)	59 (14.18)	<0.001
Back and joint pain	12 (10)	90 (29.90)	46 (45.09)	177 (42.55)	<0.001
Sore throat	32 (26.67)	46 (15.28)	26 (25.49)	219 (52.64)	<0.001
Runny nose	44 (36.9)	15 (4.98)	17 (16.7)	172 (41.34)	<0.001
Sneezing	30 (25)	6 (1.99)	12 (11.8)	114 (27.40)	<0.001

Table S2: Frequency of self-reported auditory symptoms among patients with COVID-19 during the acute phase of the disease

**Auditory symptoms**	**Un-infected (N=120) N (%)**	**Alpha & Beta (N=301) N (%)**	**Delta variant (N=77) N (%)**	**Omicron variant (N=416) N (%)**	***P*-value**
Before COVID 19					
Hearing loss	5 (4.17)	23 (7.64)	6 (5.88)	19 (4.57)	0.224
Tinnitus	4 (3.33)	9 (2.99)	8 (7.84)	16 (3.5)	0.191
After COVID 19					
Hearing loss					0.010
Mild	2 (1.67)	14 (4.65)	11 (10.78)	37 (8.89)	
Moderate	0	4 (1.33)	0	3 (0.72)	
Severe	0	1 (0.33)	1 (0.98)	0	
Total	2 (1.67)	19 (6.31)	12 (11.76)	40 (9.61)	
Tinnitus					0.005
Mild	2 (1.67)	23 (7.64)	10 (9.80)	48 (11.53)	
Moderate	0	5 (1.66)	3 (2.94)	3 (0.72)	
Severe	0	2 (0.66)	1 (0.98)	1 (0.24)	
Total	2 (1.67)	30 (9.97)	14 (13.72)	52 (12.5)	
Aural fullness					0.002
Mild	8 (6.67)	43 (14.28)	16 (15.69)	84 (20.19)	
Moderate	0	10 (3.32)	10 (9.80)	3 (0.72)	
Severe	0	4 (1.33)	1 (0.98)	1 (0.24)	
Total	9 (7.5)	57 (18.93)	27 (26.47)	88 (21.15)	

Table S3: Multivariate logistic binary regression analysis for hearing loss reported during the acute phase of COVID-19 disease.

	B	S.E.	Wald	df	*P*-value	OR	95% CI for OR	
							Lower	Upper
Group (R: Control)			5.635	3	.131			
Alpha/Beta	.794	.811	.958	1	.328	2.212	.451	10.845
Delta	1.293	.830	2.427	1	.119	3.643	.716	18.531
Omicron	1.427	.759	3.536	1	.060	4.168	.941	18.453
Gender (R: Male)	-.563	.287	3.863	1	.049	.569	.325	.998
Age	-.001	.014	.009	1	.925	.999	.971	1.027
Diabetes	.027	.540	.002	1	.961	1.027	.357	2.958
Hypertension	.281	.478	.346	1	.557	1.325	.519	3.383
Respiratory diseases	.696	.428	2.642	1	.104	2.006	.867	4.644
Anemia	.606	.449	1.820	1	.177	1.833	.760	4.423
Cough	.254	.301	.708	1	.400	1.289	.714	2.327
Breathing difficulties	-.113	.318	.127	1	.721	.893	.479	1.665
Fatigue	-.009	.310	.001	1	.976	.991	.540	1.818
Fever	-.081	.289	.078	1	.780	.922	.524	1.624
Headache	.375	.286	1.722	1	.189	1.455	.831	2.547
Migraine	1.088	.410	7.039	1	.008	2.968	1.329	6.630
Loss of smell	.999	.397	6.340	1	.012	2.716	1.248	5.912
Loss of taste	-.296	.420	.495	1	.482	.744	.326	1.696
Chest pain	.028	.362	.006	1	.938	1.028	.506	2.089
Diarrhea	-.452	.377	1.433	1	.231	.636	.304	1.334
Back & joint pain	.814	.297	7.524	1	.006	2.257	1.262	4.037
Sore throat	-.016	.310	.003	1	.959	.984	.536	1.806
Runny nose	-.084	.344	.059	1	.808	.920	.469	1.804
Sneezing	.013	.374	.001	1	.973	1.013	.486	2.109
Antiviral drug	-.097	.231	.176	1	.675	.908	.577	1.428
Constant	-4.234	.889	22.661	1	.000	.014		

Table S4: Multivariate logistic binary regression analysis for tinnitus reported during the acute phase of COVID-19 disease.

	B	S.E.	Wald	df	*P*-value	OR	95% CI for OR	
							Lower	Upper
Group (R: Control)			4.089	3	.252			
Alpha/Beta	1.461	.788	3.435	1	.064	4.312	.919	20.223
Delta	1.487	.809	3.374	1	.066	4.422	.905	21.605
Omicron	1.518	.751	4.079	1	.043	4.561	1.046	19.887
Gender (R: Male)	.135	.253	.287	1	.592	1.145	.698	1.878
Age	.027	.012	4.760	1	.029	1.027	1.003	1.052
Diabetes	.225	.412	.298	1	.585	1.252	.558	2.811
Hypertension	.638	.379	2.831	1	.092	1.893	.900	3.982
Respiratory diseases	.566	.387	2.143	1	.143	1.761	.826	3.757
Anemia	.506	.404	1.569	1	.210	1.659	.752	3.660
Cough	.019	.261	.005	1	.942	1.019	.611	1.700
Breathing difficulties	.139	.269	.266	1	.606	1.149	.678	1.947
Fatigue	.355	.272	1.704	1	.192	1.426	.837	2.430
Fever	-.016	.255	.004	1	.950	.984	.597	1.622
Headache	.383	.250	2.356	1	.125	1.467	.899	2.394
Migraine	.861	.383	5.065	1	.024	2.366	1.118	5.009
Loss of smell	.817	.361	5.125	1	.024	2.264	1.116	4.595
Loss of taste	-.885	.395	5.008	1	.025	.413	.190	.896
Chest pain	.312	.299	1.089	1	.297	1.366	.761	2.453
Diarrhea	-.048	.306	.024	1	.876	.953	.523	1.737
Back & joint pain	.572	.259	4.877	1	.027	1.772	1.067	2.945
Sore throat	-.470	.283	2.750	1	.097	.625	.359	1.089
Runny nose	.172	.310	.308	1	.579	1.188	.647	2.181
Sneezing	.310	.331	.877	1	.349	1.364	.712	2.610
Antiviral drug	-.176	.193	.831	1	.362	.838	.574	1.225
Constant	-5.536	.865	40.913	1	.000	.004		

Table S5: Multivariate logistic binary regression analysis for aural fullness reported during the acute phase of COVID-19 disease.

	B	S.E.	Wald	df	*P*-value	OR	95% CI for OR	
							Lower	Upper
Group (R: Control)			2.755	3	.431			
Alpha/Beta	.549	.425	1.672	1	.196	1.732	.753	3.983
Delta	.744	.452	2.705	1	.100	2.104	.867	5.107
Omicron	.458	.394	1.352	1	.245	1.581	.731	3.419
Gender (R: Male)	.222	.195	1.292	1	.256	1.248	.852	1.830
Age	-.002	.010	.036	1	.850	.998	.979	1.017
Diabetes	.001	.369	.000	1	.998	1.001	.486	2.064
Hypertension	-.374	.385	.943	1	.332	.688	.324	1.463
Respiratory diseases	.246	.343	.513	1	.474	1.279	.653	2.505
Anemia	.216	.346	.391	1	.532	1.242	.630	2.448
Cough	.059	.202	.085	1	.770	1.061	.714	1.577
Breathing difficulties	-.117	.218	.288	1	.591	.890	.581	1.363
Fatigue	.456	.209	4.756	1	.029	1.578	1.047	2.378
Fever	.068	.199	.116	1	.734	1.070	.724	1.581
Headache	.231	.197	1.368	1	.242	1.259	.856	1.854
Migraine	.415	.326	1.624	1	.203	1.515	.800	2.868
Loss of smell	.572	.289	3.928	1	.047	1.772	1.006	3.122
Loss of taste	-.064	.300	.045	1	.832	.938	.521	1.689
Chest pain	.325	.247	1.734	1	.188	1.384	.853	2.243
Diarrhea	.017	.241	.005	1	.943	1.017	.634	1.633
Back & joint pain	.576	.199	8.359	1	.004	1.780	1.204	2.630
Sore throat	.195	.214	.835	1	.361	1.216	.800	1.848
Runny nose	-.225	.246	.835	1	.361	.798	.493	1.294
Sneezing	.043	.264	.027	1	.871	1.044	.622	1.751
Antiviral drug	-.133	.161	.684	1	.408	.875	.639	1.200
Constant	-2.791	.503	30.801	1	.000	.061		

Table S6: Frequency of self-reported vestibular symptoms among patients with COVID-19 during the acute phase of the disease

**Vestibular symptoms**	**Un-infected (N=120) N (%)**	**Alpha & Beta (N=301) N (%)**	**Delta variant (N=50) N (%)**	**Omicron variant (N=416) N (%)**	***P*-value**
Before COVID 19					
Imbalance	1 (0.833	10 (3.32)	0	5 (1.20)	0.054
Dizziness	1 (0.83)	19 (6.31)	5 (4.90)	8 (1.92)	0.004
Vertigo	1 (0.83)	11 (3.65)	2 (1.96)	5 (1.20)	0.097
After COVID 19					
Imbalance					0.001
Mild	1 (0.83)	18 (5.98)	13 (12.75)	46 (11.06)	
Moderate	0	5 (1.66)	0	6 (1.44)	
Severe	0	2 (0.66)	0	0	
Total	1 (0.83)	25 (8.30)	13 (12.75)	52 (12.5)	
Dizziness					<0.001
Mild	5 (4.17)	57 (18.93)	30 (29.41)	113 (27.16)	
Moderate	0	24 (7.97)	2 (1.96)	23 (5.53)	
Severe	0	9 (2.90)	3 (2.94)	0	
Total	5 (4.17)	90 (29.90)	35 (34.31)	136 (32.69)	
Vertigo					< 0.001
Mild	1 (0.83)	47 (15.61)	28 (27.45)	99 (32.79)	
Moderate	0	17 (5.65)	2 (1.96)	19 (4.57)	
Severe	0	9 (2.99)	0	2 (0.48)	
Total	1 (0.83)	73 (24.25)	30 (29.41)	121 (29.1)	

Table S7: Multivariate logistic binary regression analysis for unsteadiness reported during the acute phase of COVID-19 disease.

	B	S.E.	Wald	df	*P*-value	OR	95% CI for OR	
							Lower	Upper
Group (R: Control)			4.293	3	.231			
Alpha/Beta	2.124	1.066	3.974	1	.046	8.368	1.036	67.555
Delta	2.162	1.077	4.032	1	.045	8.688	1.053	71.679
Omicron	2.125	1.033	4.236	1	.040	8.373	1.107	63.353
Gender (R: Male)	.297	.262	1.283	1	.257	1.345	.805	2.247
Age	.012	.013	.818	1	.366	1.012	.987	1.037
Diabetes	-1.050	.577	3.307	1	.069	.350	.113	1.085
Hypertension	.562	.421	1.780	1	.182	1.754	.768	4.005
Respiratory diseases	-.013	.442	.001	1	.977	.987	.415	2.347
Anemia	.288	.425	.458	1	.499	1.334	.579	3.070
Cough	.593	.282	4.406	1	.036	1.809	1.040	3.145
Breathing difficulties	-.042	.274	.024	1	.877	.958	.560	1.641
Fatigue	.728	.285	6.527	1	.011	2.070	1.185	3.618
Fever	-.130	.257	.256	1	.613	.878	.530	1.454
Headache	.210	.254	.684	1	.408	1.233	.750	2.027
Migraine	.180	.418	.185	1	.667	1.197	.527	2.719
Loss of smell	-.106	.393	.073	1	.787	.899	.416	1.942
Loss of taste	.059	.404	.022	1	.883	1.061	.481	2.342
Chest pain	.020	.309	.004	1	.948	1.020	.556	1.871
Diarrhea	-.104	.311	.112	1	.738	.901	.490	1.656
Back & joint pain	.477	.259	3.397	1	.065	1.612	.970	2.678
Sore throat	-.053	.280	.035	1	.851	.949	.548	1.644
Runny nose	.016	.315	.003	1	.959	1.016	.548	1.886
Sneezing	.175	.333	.277	1	.598	1.192	.621	2.288
Antiviral drug	-.011	.193	.003	1	.954	.989	.677	1.444
Constant	-5.923	1.131	27.452	1	.000	.003		

Table S8: Multivariate logistic binary regression analysis for dizziness reported during the acute phase of COVID-19 disease.

	B	S.E.	Wald	df	*P*-value	OR	95% CI for OR	
							Lower	Upper
Group (R: Control)			15.419	3	.001			
Alpha/Beta	2.000	.515	15.080	1	.000	7.386	2.692	20.263
Delta	1.772	.536	10.951	1	.001	5.883	2.060	16.805
Omicron	1.562	.488	10.227	1	.001	4.768	1.831	12.418
Gender (R: Male)	.441	.180	6.029	1	.014	1.554	1.093	2.209
Age	.017	.009	3.760	1	.052	1.017	1.000	1.035
Diabetes	-.112	.312	.130	1	.719	.894	.485	1.648
Hypertension	-.329	.322	1.044	1	.307	.720	.383	1.352
Respiratory diseases	-.049	.323	.023	1	.879	.952	.506	1.793
Anemia	.637	.320	3.975	1	.046	1.891	1.011	3.538
Cough	.130	.183	.504	1	.478	1.139	.795	1.631
Breathing difficulties	.290	.194	2.238	1	.135	1.336	.914	1.954
Fatigue	.788	.185	18.219	1	.000	2.198	1.531	3.156
Fever	.267	.179	2.226	1	.136	1.305	.920	1.853
Headache	.445	.175	6.440	1	.011	1.561	1.107	2.202
Migraine	.243	.317	.590	1	.443	1.275	.685	2.373
Loss of smell	.204	.267	.586	1	.444	1.227	.727	2.070
Loss of taste	-.073	.275	.071	1	.790	.930	.542	1.593
Chest pain	-.018	.229	.006	1	.938	.982	.627	1.539
Diarrhea	-.210	.222	.892	1	.345	.811	.524	1.253
Back & joint pain	.305	.180	2.856	1	.091	1.356	.952	1.931
Sore throat	.307	.193	2.530	1	.112	1.359	.931	1.984
Runny nose	-.050	.224	.051	1	.822	.951	.613	1.475
Sneezing	.257	.241	1.138	1	.286	1.294	.806	2.076
Antiviral drug	-.138	.137	1.013	1	.314	.871	.666	1.140
Constant	-4.538	.581	61.080	1	.000	.011		

Table S9: Multivariate logistic binary regression analysis for vertigo reported during the acute phase of COVID-19 disease.

	B	S.E.	Wald	df	*P*-value	OR	95% CI for OR	
							Lower	Upper
Group (R: Control)			9.398	3	.024			
Alpha/Beta	3.168	1.034	9.398	1	.002	23.767	3.135	180.173
Delta	3.080	1.045	8.686	1	.003	21.759	2.806	168.731
Omicron	3.030	1.019	8.834	1	.003	20.695	2.806	152.611
Gender (R: Male)	.487	.190	6.561	1	.010	1.627	1.121	2.362
Age	.015	.009	2.415	1	.120	1.015	.996	1.034
Diabetes	.318	.332	.922	1	.337	1.375	.718	2.634
Hypertension	-.603	.369	2.671	1	.102	.547	.266	1.128
Respiratory diseases	-.071	.346	.042	1	.838	.932	.472	1.837
Anemia	.670	.331	4.084	1	.043	1.954	1.020	3.742
Cough	-.107	.195	.299	1	.585	.899	.613	1.318
Breathing difficulties	.217	.203	1.142	1	.285	1.242	.835	1.849
Fatigue	.699	.198	12.401	1	.000	2.011	1.363	2.967
Fever	-.035	.192	.033	1	.856	.966	.663	1.407
Headache	.565	.187	9.107	1	.003	1.760	1.219	2.540
Migraine	.276	.324	.725	1	.394	1.317	.699	2.484
Loss of smell	.412	.278	2.206	1	.137	1.510	.877	2.603
Loss of taste	.258	.283	.830	1	.362	1.295	.743	2.256
Chest pain	.233	.237	.971	1	.324	1.263	.794	2.007
Diarrhea	-.182	.234	.604	1	.437	.833	.526	1.319
Back & joint pain	.348	.191	3.314	1	.069	1.416	.974	2.058
Sore throat	.159	.206	.594	1	.441	1.172	.783	1.755
Runny nose	-.176	.239	.545	1	.460	.838	.525	1.339
Sneezing	.274	.256	1.142	1	.285	1.315	.796	2.171
Antiviral drug	-.226	.155	2.132	1	.144	.798	.589	1.080
Constant	-5.876	1.070	30.166	1	.000	.003		

Table S10: Univariate logistic binary regression analysis for auditory and vestibular symptoms reported during the acute phase of COVID-19 variants while considering only un-infected controls who self-reported contracting the seasonal flu (reference group: un-infected controls with seasonal flu).

	OR	95% CI for OR		*P*-Value
		Lower	Upper	
Hearing loss				
Alpha/beta	1.99	0.45	8.77	.364
Delta	3.93	0.85	18.21	.080
Omicron	3.14	0.74	13.33	.121
Tinnitus				
Alpha/beta	3.27	0.76	14.05	0.112
Delta	4.69	1.03	21.41	0.046
Omicron	4.21	1.00	17.77	0.050
Aural Fullness				
Alpha/beta	1.35	0.63	2.89	0.442
Delta	2.08	0.90	4.79	0.085
Omicron	1.55	0.74	3.27	0.249
Unsteadiness				
Alpha/beta	5.44	0.72	40.89	0.100
Delta	8.76	1.12	68.77	0.039
Omicron	8.57	1.16	63.18	0.035
Dizziness				
Alpha/beta	4.777	1.852	12.322	0.001
Delta	5.851	2.148	15.936	0.001
Omicron	5.440	2.130	13.891	0.0001
Vertigo				
Alpha/beta	19.211	2.616	141.060	.004
Delta	25.000	3.311	188.761	.002
Omicron	24.324	3.333	177.515	.002
